# Smoking cessation on the African continent: Challenges and opportunities

**DOI:** 10.7196/AJTCCM.2019.v25i2.015

**Published:** 2019-07-31

**Authors:** C Batini, T Ahmed, S Ameer, G Kilonzo, O B Ozoh, R N van Zyl-Smit

**Affiliations:** 1 Department of Health Sciences, University of Leicester, UK; 2 Division of Pulmonology and UCT Lung Institute, Department of Medicine and Groote Schuur Hospital, Cape Town, South Africa; 3 Department of Medicine, Harare Central Hospital, Harare, Zimbabwe; 4 Department of Psychiatry, Hubert Kairuki Memorial University, Dar es Salaam, Tanzania; 5 Department of Medicine, College of Medicine, University of Lagos, Lagos, Nigeria

**Keywords:** smoking, cessation, South Africa

## Abstract

Tobacco smoking is one of the world’s single biggest preventable causes of death. Over 8 million people die each year of a tobacco-related illness – both directly and as a result of second-hand smoke. Combating this epidemic requires commitment from policy makers, healthcare workers and civil society. The WHO has invested extensively in supporting policy frameworks to assist countries to combat tobacco advertising, sales and promotion. Despite these interventions, over 1 billion people actively smoke, of whom >80% live in low- or middle-income countries. Strong policies, high taxation and cigarette pricing dissuade smokers effectively, but the clinician is frequently the individual who is faced with the smoker wishing to quit. Although many African countries have policies regarding tobacco control, very few have programmes to support smokers who wish to quit, and even fewer have active training programmes to equip healthcare practitioners to assist active smokers in breaking their addiction to nicotine. We present a perspective from several countries across the African continent, highlighting the challenges and opportunities to work together to build capacity for smoking cessation services throughout Africa.

## Background

The African continent produces more than 700 000 tons of tobacco yearly, the three highest producers being Zimbabwe, Malawi and Tanzania.^[1]^ The world bank estimates that 80% of the world’s ~1 billion smokers live in low- and low-middle-income countries. The WHO Framework Convention on Tobacco Control (FCTC) and the MPOWER strategy, instituted in 2005, have assisted governments in tobacco control efforts.^[2]^ Tobacco control efforts via **M**onitoring tobacco use, **P**rotecting people from tobacco use, **O**ffering help to quit, **W**arning about the dangers of tobacco, **E**nforcing bans on advertising and sponsorship and **R**aising taxes on tobacco, are gaining traction globally. It is estimated that roughly two-thirds of the world’s population is protected by at least one MPOWER measure.^[2]^ The uptake of each measure varies across continents and countries, but a striking discrepancy is seen in low- vs. high-income countries, where cost, governmental buy-in and resources impact on which measures are implementable.

The MPOWER strategy that is reported to be the most underutilised is that of offering help to quit tobacco use. Article 14 of the FCTC promotes tobacco cessation awareness and support for tobacco dependence, including that nicotine replacement therapy (NRT) is on the WHO essential drug list. Despite this, the 2017 WHO Report on the Global Tobacco Epidemic detailed that ‘less than one-third of high-income countries offer complete cessation support, as do fewer than one in 10 middle-income countries, with only one low-income country (Senegal) offering full cessation support.’ It is estimated that only 15% of the world’s population have access to appropriate cessation support.^[3]^

On the African continent, the establishment of effective tobacco cessation services is hampered by multiple challenges. These range from lack of training for healthcare providers, competing disease priorities, e.g. HIV and TB, lack of access to affordable medications and lack of data and locally applicable tobacco cessation guidelines.^[3,4]^

A fledgling group of interested parties established the African Collaborative Consortium to Evaluate Strategies to Stop Smoking (ACCESSS). The aim of the consortium is to increase awareness of smoking cessation as an effective strategy to address the tobacco epidemic. Additionally, to cultivate a group of clinicians around Africa gathering local data to support locally appropriate cessation strategies and to educate fellow clinicians and students in smoking cessation strategies. Although each country faces its own unique challenges, many of the factors are likely to be similar across low- and middle-income countries. Likewise, each country will afford different opportunities to support and lead tobacco cessation based on their size, location and experience.

A simple statistic such as smoking prevalence is not available for all countries within Africa. The 2017 WHO report on the global tobacco epidemic is missing any data on a third of countries on the continent, as well as missing complete data for many other countries.^[2]^ In this article, we present a perspective from 5 African countries to highlight some of the issues and information gaps across the continent relating to smoking cessation.

To our knowledge, South Africa is the only country with specific smoking cessation guidelines in Africa.^[5]^ Although there are strong bans on tobacco advertising, smoke-free buildings and warnings on packaging, tobacco smoking is estimated to cause more than 8% of all deaths, increasing the risk for mortality from tuberculosis (odds ratio (OR) 1.61), chronic obstructive pulmonary disease (COPD) (OR 2.5), lung cancer (OR 4.8) and ischaemic heart disease (OR 1.7).^[6]^ The most recent estimates for SA are that 37% of men and 6.8% of women smoke. This ranges from 42.9% of men and 25.3% of women in the Western Cape region to 26.4% and 1.3%, respectively, in Limpopo province.^[7]^ Only a single government facility smoking cessation clinic exists in the country. Medical schools do not actively teach smoking cessation approaches, and healthcare workers are ill-equipped to support smokers wishing to quit. However, there are some initiatives with Quit lines (National Council Against Smoking), and web-based platforms for smoking cessation (CANSA association).

Nigeria, the most populous country in Africa, with a population of 198 million, has had relatively low and stable smoking rates of 9.3% among adults (≥15 years).^[8]^ Although specific population groups such as commercial drivers and out-of-school adolescents/ youths have much higher tobacco smoking rates of ~30%.^[9,10]^ The Nigerian Tobacco Control Alliance (NTCA) is working in partnership with the FTCT to implement the Nigerian Tobacco Control Act, which was passed into law in 2015. Despite this, there is very limited availability of smoking cessation clinics in Nigeria and most tertiary hospitals do not have smoking cessation clinics. A recent study based on patients’ chart review reports that only about 12% of current smokers had documentation of being given any form of smoking cessation advice.^[11]^ This stems from poor knowledge and competence in providing smoking cessation service among healthcare workers, mainly due to lack of formal training in this regard.^[12,13]^ Furthermore, the absence of smoking cessation clinics hinders referrals for the service when healthcare workers lack competence. Pharmacotherapy for smoking cessation is scarce in Nigeria and only NRT may be found in a few pharmacies.

In Sudan, one of the most geographically diverse states in Africa with a population of ~40 million people, there are limited data on the trends for tobacco consumption. The best estimates for smoking among men and women are 1.3% and 0.8%, respectively, but youth smoking is estimated at 14.5% of males and 7.3% of females.^[2]^ Although signatory to the FCTC in 2005, very few of the policies have been effectively implemented. The National Tobacco Control Initiative (NTCI) has been established to implement tobacco control strategies in Sudan. Currently there are no smoking cessation services available nor access to NRT.

In Zimbabwe, the world’s 6th largest producer of tobacco, smoking is rare among women aged 15 - 49, with <1% reporting that they currently smoke cigarettes.^[14]^ Among men aged 15 - 49 years, 17% currently smoke tobacco.^[14]^ Zimbabwe undertook accession to be an FCTC partner on 4 December 2014. There are currently designated smoking rooms in healthcare facilities, educational facilities, including universities, and in public transport but no such facilities in restaurants, offices, cafés, pubs and bars. Zimbabwe has small or no warnings on tobacco products and no ban on advertising tobacco products.^[15]^

There are currently no services for smoking cessation in the Zimbabwean public sector, including a telephone quit line or coverage of smoking cessation products.^[2]^ There are no smoking cessation products on the essentials drug list, but in the private sector one can access smoking cessation in some private healthcare professional offices.^[2]^ There is pharmacotherapy available but at very high cost (NRT 130 USD/week; varenicline 250 USD/month). There are no specific smoking cessation guidelines or a national smoking cessation strategy or even a programme for primary care workers, and smoking cessation does not need to be recorded in the medical records.^[2]^

Based on WHO figures for Tanzania in 2016, 12.4% of males and 2.2% of male youths were smokers, while 8.8% of females and 1.1% of female youths were current smokers.^[2]^ Taxation reaches 47.2% (excise tax plus sales tax) but is not aggressively pursued as a means to discourage tobacco use. Selling tobacco products to minors is legally prohibited. Health warnings are required on tobacco products and there is a ban on advertising in mass media, including billboards.^[15]^ Health education against smoking is a prominent form of control and is carried out by various organisations, including the National Anti-tobacco Committee formed by the Ministry of Health.

In Tanzania, there have not been any attempts to study the applicability of standard smoking cessation approaches. There are anecdotal accounts of few clients who have benefited from personalised psychosocial counselling combined with NRT. Smoking cessation is largely integrated with clinical work in general, focusing on patients with conditions that are caused or worsened by smoking such as cardiac, respiratory and mental conditions. In private clinics and at the main teaching hospital in Dar es Salaam, NRT is available. Smoking cessation has also been integrated into the methadone clinic at Muhimbili National Hospital in Dar es Salaam.

## Discussion

The tobacco industry has been targeting emerging markets and especially vulnerable populations outside of America and Europe as these markets decline.^[15]^ In particular, sub-Saharan Africa, has seen >50% increase in tobacco consumption from 1980 to 2016.^[15]^ It is clear that Africa is not suitable to a ‘one-stop-shop approach’ to tobacco cessation given the complexities of the cultural, economic, tobacco prevalence and available resources admixture. It is also evident that the FCTC policies regarding monitoring, advertising, and smoking bans have been adopted and effectively implemented in several countries. Despite this, the capacity to undertake effective smoking cessation is limited by poor availability of basic services such as quit lines, and/or basic self-funded NRT availability.

Effective treatment strategies for nicotine addiction without policies to reduce tobacco uptake and availability are likely to have limited impact on the epidemic. Similarly, policy on its own does very little to assist highly addicted smokers to quit but even potentially worsens the situation for a heavily addicted smoker, with high costs of cigarettes and restricted areas in which to smoke. A combined strategy of effective and stringent tobacco control along with effective options to assist smokers in overcoming their addiction to nicotine is likely to be more effective for overall tobacco control. In turn, this will reduce the number of active smokers as well as the burden of smoking-related illness on the healthcare systems that can least afford it.

## Conclusion

It is hoped that by promoting tobacco cessation research and training, and equipping clinicians across the continent, locally driven and relevant interventions can be instituted. All healthcare workers need the skills and capacity to effectively engage with active smoking behaviour to reduce the burden and cost to healthcare systems. The ‘missing’ FCTC: ‘Article 14’ needs to be activated alongside the national legislative tobacco control policies. Preventing, discouraging and stopping active smoking together, will have the greatest impact on the tobacco epidemic and hopefully reverse the current trends to greater tobacco consumption and related illness across many parts of the African continent.
